# Cooperation and coordination in heterogeneous populations

**DOI:** 10.1098/rstb.2021.0504

**Published:** 2023-05-08

**Authors:** Xiaomin Wang, Marta C. Couto, Nianyi Wang, Xinmiao An, Bin Chen, Yali Dong, Christian Hilbe, Boyu Zhang

**Affiliations:** ^1^ Laboratory of Mathematics and Complex Systems, Ministry of Education, School of Mathematical Sciences, Beijing Normal University, Beijing 100875, People’s Republic of China; ^2^ School of Environment, Beijing Normal University, Beijing 100875, People’s Republic of China; ^3^ School of Systems Science, Beijing Normal University, Beijing 100875, People’s Republic of China; ^4^ Max Planck Research Group Dynamics of Social Behavior, Max Planck Institute for Evolutionary Biology, Plön 24306, Germany

**Keywords:** cooperation, coordination, threshold public goods game, inequality, asymmetric game, evolutionary game theory

## Abstract

One landmark application of evolutionary game theory is the study of social dilemmas. This literature explores why people cooperate even when there are strong incentives to defect. Much of this literature, however, assumes that interactions are symmetric. Individuals are assumed to have the same strategic options and the same potential pay-offs. Yet many interesting questions arise once individuals are allowed to differ. Here, we study asymmetry in simple coordination games. In our set-up, human participants need to decide how much of their endowment to contribute to a public good. If a group’s collective contribution reaches a pre-defined threshold, all group members receive a reward. To account for possible asymmetries, individuals either differ in their endowments or their productivities. According to a theoretical equilibrium analysis, such games tend to have many possible solutions. In equilibrium, group members may contribute the same amount, different amounts or nothing at all. According to our behavioural experiment, however, humans favour the equilibrium in which everyone contributes the same proportion of their endowment. We use these experimental results to highlight the non-trivial effects of inequality on cooperation, and we discuss to which extent models of evolutionary game theory can account for these effects.

This article is part of the theme issue ‘Half a century of evolutionary games: a synthesis of theory, application and future directions'.

## 1. Introduction

Fifty years after the introduction of ‘evolutionarily stable strategies’ [1,2], evolutionary game theory has become a powerful toolbox to analyse evolutionary processes and human behaviours. This toolbox can be applied to a wide variety of questions. Using evolutionary game theory, researchers have explored how conventions evolve [3], how people learn from others [4] and how recurrent corruption threatens the stability of societies [5]. Perhaps one of the most prominent applications of evolutionary game theory is the study of cooperation [6,7]. This field aims to describe which mechanisms promote cooperative behaviours, and in which social and ecological environments cooperation is most likely to spread. Among the mechanisms that facilitate cooperation are kin selection [8,9] and group selection [10,11], direct [12–14] and indirect reciprocity [15,16], network structure [17–19], and reward and punishment [20–23].

Models of cooperation typically assume that all members of an evolving population are symmetric. That is, all individuals have the same sets of strategies and identical incentives to choose each strategy. Yet in most human and animal populations, inequality is ubiquitous. People frequently differ in their endowments, productivities, their shares of rewards, and the positions they occupy in social hierarchies [24,25]. Such inequalities gives rise to asymmetric games, where individuals differ in what they can do, and what consequences their actions have. In the context of cooperation, such asymmetric games allow for a range of new exciting questions. For example, how do different kinds of inequality affect the way people cooperate? Also, how do these inequalities influence human notions of fairness?

In this paper, we discuss some of these questions in the context of a simple coordination game, the threshold public goods game. This game has many possible equilibria, but group members disagree on which equilibrium they prefer. To explore the effect of exogenous inequality, we consider two independent sources of asymmetry. Individuals can either differ in their endowments (i.e. their wealth) or in their productivities (i.e. the efficiency of their contributions to the public good). We use this set-up to explore how inequality impedes overall coordination (if at all), and how it affects which equilibria are chosen. We address these questions with both a theoretical analysis and a behavioural experiment.

As a part of this analysis, we also ask to which extent classical evolutionary dynamics can accurately predict the behaviours that emerge in the experiment. Perhaps somewhat surprisingly, we find that a naive application of evolutionary models fails to reproduce many of the empirical patterns. For example, models based on replicator dynamics with random initial conditions tend to overestimate how often people defect, and they underestimate how often less productive individuals cooperate. However, we also show that predictions become more accurate when initial conditions match the empirical first-round behaviours. These results suggest that especially in games with natural focal points, a naive application of evolutionary game dynamics may mis-predict which equilibrium is most likely to occur. These predictions improve dramatically, however, once the participants’ true initial play is taken into account.

Overall, the contribution of our study is twofold. First, we provide novel empirical evidence on how people coordinate in the presence of exogenous asymmetries. Second, our data also allow us to test the predictive power of different types of evolutionary dynamics. Both contributions may help to further improve existing models on the evolution of cooperation in asymmetric social dilemmas.

## 2. An overview of the previous literature on asymmetric public goods games

Traditionally, a classical model to study the effect of inequality on cooperation is the linear public goods game [26,27]. In this game, individuals decide how much of their endowment to contribute to a public good. All contributions are multiplied by some factors, and the resulting amount is evenly shared among all group members. In asymmetric public goods games, subjects can differ in their endowments, productivities and in how much they benefit from public goods. For one-shot games, it has been shown that heterogeneity can have a positive effect on cooperation—players with higher productivity and share of reward tend to contribute all their endowment [28,29] (Zhang B, Dong Y, Qin C-Z, Gavrilets S, 2022, Kinship can hinder cooperation in heterogeneous populations, unpublished manuscript). If the public goods game is played repeatedly, the effect of heterogeneity on cooperation is more complex. Although extreme inequality prevents cooperation, slightly unequal endowments may be necessary for cooperation to evolve if players also differ in other dimensions [26].

In general, however, the interaction of inequality and cooperation is non-trivial. A vast amount of experimental studies observe that endowment inequality tends to reduce cooperation even in the one-shot public goods game [30–32]. Furthermore, the relative contributions of the rich are often lower than those of the poor, but their absolute contributions tend to be higher [33–35]. Compared with asymmetric endowments, asymmetric productivities have a neutral or positive impact on contributions, and highly productive players often contribute more than players with a low productivity [36–38]. By contrast, the effect of asymmetric sharing of public goods seems to be neutral, and players often contribute in proportion to their share [26,39–42]. Finally, when there are both endowment and productivity heterogeneity, cooperation is maximized if the two sources of heterogeneity are aligned, such that more productive individuals have higher endowment [26]. By contrast, cooperation quickly breaks down if endowments and productivities are misaligned. Similarly, also a combination of endowment and reward heterogeneity can increase cooperation [43]. Finally, the joint effect of productivity and reward inequalities on cooperation is not significant [40].

While in the linear public goods game, the dominant action in a single game is to defect, social dilemmas can also arise in games in which cooperation is an equilibrium. One example is the threshold public goods game [44–46]. Here, individuals only benefit from contributions to a public good if their contributions exceed a certain threshold. Threshold public goods games tend to have many equilibria, including a defective equilibrium in which no one contributes, and a set of cooperative equilibria in which the group’s collective contribution matches the threshold. However, different individuals may prefer different cooperative equilibria: everyone prefers the threshold to be reached, but they prefer the other group members to make the necessary contributions.

Recently, a particular variant of the threshold public goods game has attracted much attention, the climate game or collective-risk dilemma [47–53]. For this game, evolutionary game theory suggests that wealth inequality can enhance cooperation. Furthermore, in the multi-period climate game, players with higher endowments, productivities and risks of loss generally contribute more to the common pool at the cooperative equilibrium [53–56]. However, experimental studies on the asymmetric threshold public goods game and the climate game observed an adverse effect of endowment inequality on coordination [44,45,49,52]. Although rich subjects often contribute more than in the homogenous case, this surplus is often overcompensated by the accompanying decrease in contributions by the poor [52]. The negative effect of endowment inequality could be reduced by introducing additional mechanisms, such as setting an intermediate target [48], communication between players [49], and the frame of ‘contributing’ (rather than ‘keeping’) in ‘absolute’ terms (rather than ‘relative’) [46]. By contrast, productivity inequality does not seem to have a significant effect [51]. Furthermore, the effects of multiple dimensions of inequality on coordination is generally more complex [53].

Most of the previous studies on the impact of asymmetry on coordination are based on a multi-period climate game. Here, players are required to reach the target at the end of a series of repeated contributions [47–50,52–54,56]. Such a multi-period structure can give rise to complex dynamics, since individuals may first adopt a wait-and-see approach [53,54,56]. In the following, we thus explore the effect of asymmetry in a more simple setting, where each experimental round represents an independent game. We use this framework to systematically explore the effect of two sources of asymmetry, by introducing inequality in the players’ endowments and their productivities.

## 3. Model and theoretical predictions for the one-shot game

To explore the effect of exogenous inequality on coordination, we use a comparably simple set-up. We consider an asymmetric threshold public goods game among two players, player 1 and player 2. The game proceeds as follows. First, each player *i* (with *i* = 1, 2) receives a fixed positive integer endowment of *e*_*i*_. Then the players individually decide which amount *c*_*i*_ from their endowment they wish to contribute to a common pool, with *c*_*i*_ ∈ {0, 1, …, *e*_*i*_}. Each individual’s contribution is multiplied by a productivity factor *p*_*i*_. We refer to *p*_1_*c*_1_ + *p*_2_*c*_2_ as the players’ collective contribution. If the players’ collective contribution reaches a predefined threshold *θ*, each individual *i* receives a reward *r*_*i*_ in addition to their remaining endowment. Otherwise, both players only receive their remaining endowment. Therefore, pay-offs depend on the fixed parameters of the game (*e*_*i*_, *p*_*i*_, *r*_*i*_ and *θ*) and on the contribution tuple (*c*_1_, *c*_2_). The pay-off of player *i* is:

3.1 $$\pi_{i}(c_{1},c_{2})=\left\{ \begin{array}{cc} e_{i}-c_{i}+r_{i} & \text{if }p_{1}c_{1}+p_{2}c_{2}\geq \theta \\ e_{i}-c_{i}\;\; & \text{otherwise}.\;\; \end{array}\right.$$

**Figure 1.**
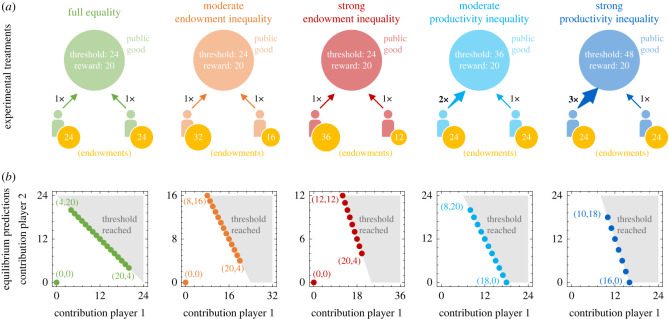
Basic set-up and predictions for a threshold public goods game. (*a*) We consider games between two players. In the beginning, players receive some fixed endowment (indicated by yellow coins). The players then independently decide how much of their endowment to contribute to a public good. The player’s effective contribution is their contribution times their individual productivity factor (indicated by the arrows). If the sum of the players’ effective contributions exceeds a threshold, both players receive a reward. We conduct experiments for five treatments. Players are either identical in all aspects, or they differ in their endowments, or they differ in their productivities. The treatment with full equality serves as our control. (*b*) To gain some insight into the logic of the game, we calculate the Nash equilibria of the one-shot game (these equilibria are marked by coloured dots). Each treatment allows for many Nash equilibria. These equilibria differ in whether or not the threshold is reached, and in how much the two players contribute. For better clarity, we highlight the most extreme Nash equilibria, by depicting the players’ respective contributions (*c*_1_, *c*_2_) in equilibrium.

For our behavioural experiment, we consider five particular instantiations of this game (figure 1*a*; electronic supplementary material, table S1). As a control, we consider a treatment with ‘full equality’. Here, individuals coincide in all dimensions, and the game is symmetric. Next, we consider two treatments in which individuals only differ in their endowments. Here, player 1 either obtains twice the endowment of player 2 (moderate endowment inequality) or three times the endowment of player 2 (strong endowment inequality). Finally, we also consider two treatments in which individuals have different productivities. Again, player 1 is either twice as productive (moderate productivity inequality), or three times as productive as player 2 (strong productivity inequality). To facilitate comparisons across treatments, the reward is kept constant, and the threshold *θ* is determined such that groups reach the threshold if both players contribute half of their endowment.

To obtain some intuitive understanding of the strategic considerations that apply to this game, we first describe the respective Nash equilibria of the one-shot (non-repeated) game (see the electronic supplementary material, S1.2 for details; in addition, S4.1 describes the possible equilibrium outcomes when the game is repeated for many rounds).

In general, the one-shot threshold public goods game can have two types of equilibria. First, there is a set of cooperative Nash equilibria in which the group’s collective contribution exactly matches the threshold. In addition, there can be a defective Nash equilibrium in which all players keep their endowment. In figure 1*b*, we illustrate the set of Nash equilibria for the five experimental treatments. Two interesting observations follow immediately. First, for the parameters of the experiment, full defection is an equilibrium under full equality and under endowment inequality, but it ceases to be an equilibrium under productivity inequality. In the last two treatments, the more productive player has a sufficiently strong incentive to contribute even if the other player defects. Second, in all treatments there are many different cooperative equilibria. In all these equilibria the group reaches the threshold, yet they differ in how the respective costs are allocated among the two players.

To further explore how participants might choose among the different cooperative equilibria, in the electronic supplementary material, S1.3 we describe a set of heuristics that participants might apply. For example, if participants strive for equal absolute contributions (EAC), they settle at the cooperative equilibrium for which *c*_1_ = *c*_2_. Similarly, if they both strive to achieve equal relative contributions (ERC), they choose the equilibrium that satisfies *c*_1_/*e*_1_ = *c*_2_/*e*_2_. In addition, participants might also strive for equal collective contributions, equal pay-offs, and a maximal collective pay-off. In the electronic supplementary material, table S2, we summarize which predictions these criteria make for each of the five treatments. As one aim of the experiment, we explore which of the above rules is best able to explain the subjects’ behaviours.

## 4. A behavioural experiment on asymmetric threshold public goods games

To explore how humans act in such threshold public goods games, we invited a total of 558 participants to interact in one of the five treatments illustrated in figure 1. In the beginning of each experimental treatment, participants are assigned to a single treatment. Each participant then plays two sets of repeated games, referred to as game 1 and game 2. At the beginning of game 1, participants are randomly matched in pairs, and randomly receive the roles of player 1 and player 2, respectively. Thereafter, they play 20 rounds of the asymmetric public goods game, during which participants keep their co-player and their respective role. After each round, participants learn each other’s contributions and pay-offs, as well as their collective contribution. In game 2, participants interact for another 20 rounds, but now with a different partner and in the opposite role (those participants who acted as player 1 in game 1 now act as player 2, and vice versa). At the end of the experiment, participants are asked to fill out a questionnaire on what they consider to be the fair contribution patterns for each of their roles. In the following, we present the results of our experiment when we combine the results of game 1 and game 2. For a detailed analysis of each separate game and more details on the experimental procedures, see the electronic supplementary material, S2.

**Figure 2.**
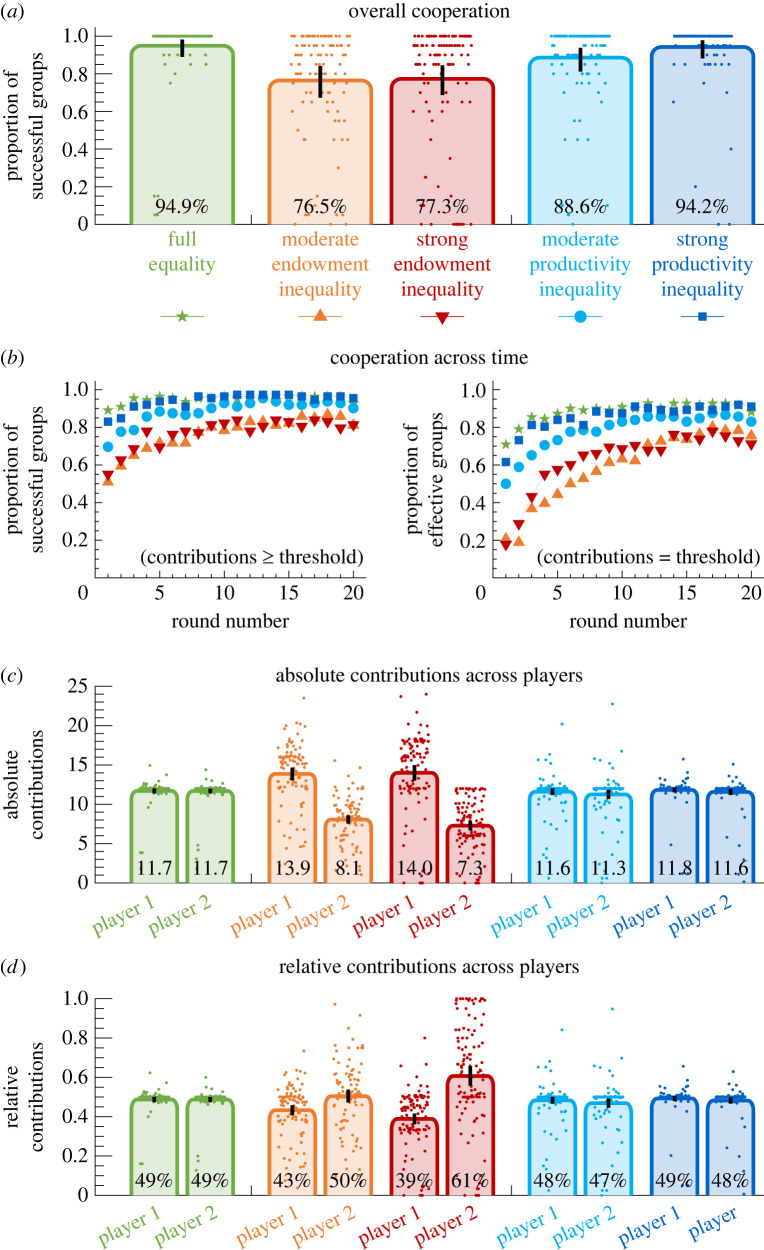
Main results of the experiment. (*a*) For each treatment, we first compute how likely groups are successful in obtaining the reward (i.e. how likely their collective contribution matches or exceeds the threshold). Dots indicate the average success rate of each individual group, averaged over all 20 rounds of the game. Compared to the treatment with full equality, both moderate and strong endowment inequality diminish a group’s success rate. In addition, also moderate productivity inequality has a weakly negative effect. (*b*) We next study the groups’ cooperation dynamics over time. To this end, we consider how often groups are successful (they match or exceed the threshold), and how often they are effective (they exactly match the threshold). In all treatments, players learn to better coordinate their contributions over time. However, in the treatments with endowment inequality, individuals find it more difficult to coordinate. (*c*,*d*) We then compare the players’ contributions across the five treatments. Dots again represent average absolute and relative contributions of the two players, averaged over the 20 rounds of the game. We observe that players contribute approximately equal amounts under full equality and under productivity inequality. In the treatments with endowment inequality, player 1 contributes more than player 2 in absolute terms (but less than player 2 relative to the players’ endowment). The error bars represent 95% confidence intervals.

To assess the effects of inequality, we consider two measures of a group’s performance. We call a group *successful* if their collective contribution matches or exceeds the threshold. Similarly, we call the group *effective* if their collective contribution exactly matches the threshold. In figure 2*a*, we compare the proportion of successful groups across the five treatments. Overall, we observe that under full equality and under productivity inequality, these proportions are largely similar, with approximately 90% of these groups meeting the threshold on average. Only when there is endowment inequality, groups become less successful (in particular, in both treatments with endowment inequality, the proportion of successful groups is significantly lower than in the treatments with productivity inequality; electronic supplementary material, table S5). To further explore these differences, we analyse how the proportion of successful groups changes over the course of the experiment. In figure 2*b*, we show that this proportion increases in all treatments, suggesting that individuals learn to better coordinate their contributions over time. However, especially in the groups with endowment inequality, it may take a substantial number of rounds until groups have learned to coordinate effectively.

In the next step, we compare the contributions of players 1 and 2 across the five treatments (figure 2*c*,*d*). Under full equality and productivity inequality, the contributions of the two players are indistinguishable. When there is endowment inequality, however, high-endowment players contribute more in absolute terms but less in relative terms (see also the electronic supplementary material, table S7). To further explore these results, we look at the groups’ contribution patterns (*c*_1_, *c*_2_) over time, separately for each treatment (see the electronic supplementary material, figure S2). When there is either full equality or productivity inequality, most participants quickly coordinate on giving half of their respective endowment. This contribution pattern is consistent with the heuristics of EAC and ERC in these treatments (see the electronic supplementary material, table S2). By contrast, in the treatments with endowment inequality, the picture is more mixed. Here, in about a quarter of groups, players continue to give half of their endowment (consistent with the ERC criterion). In addition, there is a substantial number of groups in which players either make the same absolute contribution (EAC), they contribute nothing at all, or their contribution pattern is somewhat between the patterns of ERC and EAC (see the electronic supplementary material, figure S2). Interestingly, the picture is much more clear when participants need to specify the contribution pattern they consider to be most fair. According to the post-experiment questionnaire, ERC is clearly the most common response (see the electronic supplementary material, figure S3).

In a last step, we also analyse why some groups fail to reach the threshold (see the electronic supplementary material, figure S4) and how players respond to failure (see the electronic supplementary material, figure S9). To this end, we take the outcome with ERCs as the baseline, and distinguish three categories of failing groups: (i) groups in which only player 1 contributes less than half of the endowment, (ii) groups in which only player 2 contributes less than half of the endowment, and (iii) groups in which both players fail to contribute at least half of their endowment. In the two treatments with endowment inequality (where we observe most failures), most groups either fail because player 1 (high-endowment player) falls short of contributing at least half of the endowment, or because both players contribute too little. Only in a clear minority of groups it is the low-endowment player who under-contributes. Overall, these results suggest that endowment inequality represents the biggest obstacle to coordination since no contribution pattern is a clear focal point. Finally, conditional behaviours are observed in failing groups with categories (i) and (ii), where players who contribute less than half of their endowment tend to increase their contribution in the next round (see the electronic supplementary material, figures S8 and S9). This can help to understand how players gradually coordinate on the pattern of ERCs.

## 5. Evolutionary game dynamics and equilibrium selection

When games have multiple equilibria (as in our treatments), evolutionary game theory is often interpreted as a means to single out one of these equilibria as the most salient outcome [57]. In the following, we explore to which extent classical evolutionary game dynamics predict the equilibria that arise in the experiment. To this end, we compare our experimental results to four models of evolutionary game theory. Specifically, we consider replicator dynamics [58], best-response dynamics [58], a stochastic birth–death process [59] and introspection dynamics [60]. These models have been widely used to predict human behaviour in social dilemma games [26,53,61,62]. Furthermore, we distinguish two scenarios, depending on whether individuals interpret their interactions as a series of independent one-shot games, or whether they interpret them as a repeated game (in the latter case, individuals may use conditional strategies that depend on the outcome of previous rounds). We fully discuss all these models and their results in the electronic supplementary material, S3 and S4.3. Here, we briefly summarize our results for the replicator dynamics for the one-shot game.

**Figure 3.**
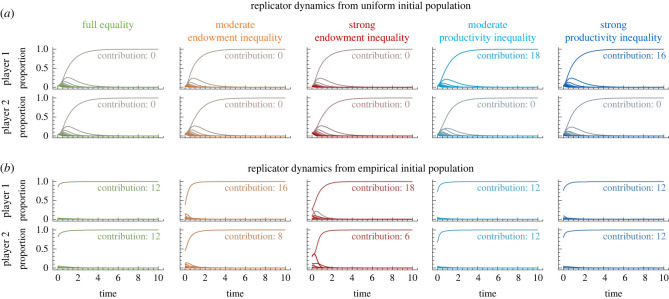
Modelling the evolution of strategies with replicator dynamics. To explore to which extent classical game dynamics are able to recover the previous empirical patterns, we consider a variety of dynamics (see the electronic supplementary material, S3 for details). Here, we report results for replicator dynamics, assuming that individuals interpret each round as an isolated one-shot game. In particular, a player’s possible strategies are all possible contributions between zero and the player’s endowment (for a repeated game analysis where players use conditional strategies, see the electronic supplementary material, S4). (*a*) First, we assume that initially, all strategies are played with equal frequency. In that case, we observe that eventually, either players do not contribute at all (full equality, endowment inequality), or that only the first player contributes (productivity inequality). (*b*) We reconsider the outcome of replicator dynamics with an initial population that matches the empirical first-round behaviour. We observe that now the model recovers the main empirical patterns. In particular, the solution according to replicator dynamics matches the most abundant experimental outcome in round 20 (see the electronic supplementary material, figure S6).

As a first approach, we assume that initially, all strategies are played with equal frequency and trace the trajectory of replicator dynamics [63,64], see figure 3*a*. The equilibria predicted by this method, however, fail to reproduce the experimental patterns. For three treatments (full equality, and both endowment inequality treatments), this method selects the mutual defection equilibrium as most salient. In the remaining two treatments (productivity inequality), replicator dynamics predicts the more productive player to make all the contributions. We find a similar mismatch between evolutionary trajectories and experimental results when we use a random sample of initial populations, or when we use different evolutionary dynamics (electronic supplementary material, figure S5). In all cases, evolutionary models underestimate the contributions of both players in the treatments with full equality and endowment inequality. In addition, they overestimate the contributions of the more productive player in the treatments with productivity inequality.

As one potential reason for this mismatch, we note that models of evolutionary game theory neglect information that is not directly represented by the pay-off matrix. This information may entail, for example, the presence of natural focal points [65]. In addition, by choosing random initial conditions, we give equal weight to all strategies. Such a choice can exaggerate the influence of dominated strategies on the subsequent dynamics. To address this issue, we have run a second set of simulations, for which we take the empirical first-round behaviour as the population’s initial strategy distribution. With this modification, replicator dynamics now predicts the most abundant experimental outcome in all five treatments (see figure 3*b*). Similarly, also the fit of all other evolutionary models is now greatly improved, as illustrated in the electronic supplementary material, figure S6. Overall, these results suggest that a naive application of classical evolutionary dynamics and learning processes may fail to anticipate the regularities of empirical play (irrespective of whether the used dynamics is deterministic or stochastic). However, evolutionary models can recover these empirical regularities when taking into account the true empirical initial distribution.

To complement this one-shot analysis, in the electronic supplementary material, S4, we provide additional analyses to capture the repeated-game character of the experiment. Among other results, we explore how individuals learn to make decisions when they can choose among all reactive strategies. When individuals use reactive strategies, they condition their behaviour on their co-player’s behaviour in the previous round. Our simulations suggest that once individuals interact repeatedly with the same partner, they are more likely to reach the threshold over time (electronic supplementary material, figure S10). At the same time, however, the repeated game analysis does not recover the empirical observation that individuals have a clear tendency to favour outcomes in which both players contribute equally (either in absolute or relative terms). These findings may be taken as another indication that focal points and the subjects’ expectations prior to the experiment may play a decisive role.

## 6. Discussion

In this paper, we study the effect of inequality in asymmetric threshold public goods games. Players decide how much of their endowment to contribute to a public good. Only if the group’s collective contribution reaches a given threshold, players receive a reward. To study the effect of exogenous inequality, individuals may either differ in their endowments, or in how productive their contributions are. In general, such threshold public goods games allow for many different equilibria. These equilibria differ in whether or not the threshold is reached, and in the relative contributions of each player.

With a behavioural experiment, we show that endowment inequality is more of an obstacle to cooperation than productivity inequality. Interestingly, the strength of asymmetry (e.g. whether one player has two times or three times the endowment of the other player) seems to have a negligible effect. Our data suggest that endowment inequality makes it particularly difficult to coordinate because different individuals may consider different contribution patterns as the most salient. While some individuals may strive to make equal absolute contributions, others may strive to equalize the individuals’ relative contributions (relative to the players’ endowments) [35,46]. Such mismatches in expectations can delay the time until individuals reach an equilibrium, or it can prevent them from reaching a cooperative equilibrium altogether. By contrast, in the treatments with full equality and productivity inequality, the two above criteria (equal absolute or relative contributions) single out the same equilibrium. This might explain why individuals find it easier to coordinate in those treatments.

Interestingly, although groups in the treatment with productivity inequality are extremely successful in reaching the threshold, they typically do not coordinate on the most efficient equilibrium. In these treatments, efficiency would require the more productive player to make all the contributions. While this equilibrium maximizes the group’s collective pay-off, it leads to a rather uneven distribution of these pay-offs, with the low-productivity player benefitting disproportionately. Instead, our experiment suggests that groups unanimously prefer the two players to make equal contributions. This observation suggests that there is an interesting efficiency dilemma on top of the usual social dilemma. Individuals may refuse to choose the most efficient equilibrium if doing so would lead to unequal pay-offs [66,67].

Using different models of evolutionary game theory, we have also explored to which extent classical evolutionary dynamics can account for these empirical regularities. To this end, we assume in the main text that individuals interpret their interactions as a series of one-shot games. This analysis does not directly mimic the set-up of the experiment, in which participants interact with the same co-player for 20 rounds. Nevertheless, in coordination games such as ours, a one-shot analysis can already give basic insights into the strategic logic of the game: in threshold public good games, individuals prefer to meet the threshold, yet they prefer their co-player to make the respective contributions. Repeated games may allow for additional equilibria. Yet the most efficient repeated-game equilibria correspond to the set of cooperative one-shot equilibria shown in figure 1 (see the electronic supplementary material, S4.1 for details).

When applied naively (i.e. when we use uniform or random initial conditions), we find that all considered evolutionary dynamics based on the one-shot game provide a poor match to the empirical data. They overestimate how often people would defect, and how often the more productive player would cooperate in the treatments with productivity inequality. One reason for this mismatch might be related to the participants’ beliefs and expectations prior to the experiment. In their daily lives, individuals rarely play any single coordination game in isolation. Rather they play a mixture of different games with different incentives to cooperate. As a result they develop simple heuristics based on inequality aversion [68,69], reference points [70], pay-off ranking [67,71] or collective pay-off maximization [66] to help them with their typical decision problems. When such individuals participate in behavioural experiments, their first-round behaviour tends to be largely affected by the heuristics they apply. In line with this view, we observe that all considered evolutionary dynamics describe the empirical patterns fairly accurately when they take the participants’ empirical first-round behaviour into account.

Overall, our study demonstrates that evolutionary game theory remains a powerful tool for modelling behavioural dynamics in asymmetric interaction. However, when predicting human behaviour in coordination games with many equilibria, they may mis-predict which equilibrium arises if some of the equilibria are more salient to participants than others. In addition, the experiments also identify another source of complexity. For instance, some players withdraw from the cooperative equilibrium by decreasing their contribution, most likely because they expect the co-player to make up for their missing contributions in the future [72,73]. In this sense, cooperation and coordination in repeated asymmetric interaction become an even more delicate issue.

## Data Availability

All data and code are available from the Dryad Digital Repository: https://doi.org/10.5061/dryad.x0k6djhnq [74]. The data are provided in the electronic supplementary material [75].
